# Extent of disease affects the usefulness of fecal biomarkers in ulcerative colitis

**DOI:** 10.1186/s12876-021-01788-4

**Published:** 2021-05-01

**Authors:** Akihito Sakuraba, Nobuki Nemoto, Noritaka Hibi, Ryo Ozaki, Sotaro Tokunaga, Oki Kikuchi, Shintaro Minowa, Tatsuya Mitsui, Miki Miura, Daisuke Saito, Mari Hayashida, Jun Miyoshi, Minoru Matsuura, Masayoshi Yoneyama, Hiroaki Ohnishi, Tadakazu Hisamatsu

**Affiliations:** 1 Department of Gastroenterology and Hepatology, Kyorin University School of Medicine, 6-20-2 Shinkawa, Mitaka-shi, Tokyo, 181-8611 Japan; 2 Department of Clinical Laboratory, Kyorin University Hospital, Tokyo, Japan; 3 Department of Laboratory Medicine, Kyorin University School of Medicine, Tokyo, Japan

**Keywords:** Disease extension, Fecal calprotectin, Fecal immunochemical test, Ulcerative colitis, Mayo endoscopic subscore

## Abstract

**Background:**

Fecal biomarkers are considered to be useful surrogate markers for endoscopic activity. Given the mechanisms of fecal biomarkers, we hypothesized that the extent of ulcerative colitis (UC; pancolitis, left-sided colitis, and proctitis) could affect the usefulness of fecal biomarkers for assessing endoscopic and clinical disease activity; however, few studies have evaluated the utility of fecal biomarkers in the disease extent of UC.

**Methods:**

Fecal calprotectin, a fecal immunochemical test for hemoglobin, and fecal lactoferrin were used as fecal biomarkers. UC patients, who underwent colonoscopy within 30 days of the fecal biomarker test, participated in this observational study. Clinical and endoscopic disease activity was assessed using the Lichtiger Index and Mayo endoscopic subscore (MES), respectively.

**Results:**

A total of 162 colonoscopies were performed on 133 UC patients. A correlation analysis between each biomarker and the MES for each disease-extent subgroup showed a decreased correlation in the proctitis compared with the other groups. With the exception of proctitis, it was possible to distinguish between MES 0 and MES ≥ 1 with high area-under-the-curve values for fecal calprotectin and fecal lactoferrin. The fecal immunochemical test for hemoglobin was superior at discriminating MES 0 for proctitis.

**Conclusions:**

For the practical application of fecal biomarkers for UC patients, it is necessary to consider disease extent before use. In particular, patients with proctitis exhibit a low correlation between stool biomarkers and endoscopic findings. The usefulness of these biomarkers for endoscopic remission is reduced, except for the fecal immunochemical test for hemoglobin.

**Supplementary Information:**

The online version contains supplementary material available at 10.1186/s12876-021-01788-4.

## Background

Ulcerative colitis (UC) is a chronic inflammatory disorder of unknown etiology. Treatment is administered to improve a patient's subjective symptoms and quality of life. Furthermore, in recent years, a treat-to-target strategy has been proposed for the purpose of improving the long-term prognosis. Endoscopic remission has been recognized as a treatment target for UC [1, 2]. Several studies have demonstrated that endoscopic remission reduces the risk of relapse [3], hospitalization [4], colectomy [5], and possibly the development of colitis-associated cancer [6] in UC patients. However, colonoscopy is a burden for patients and physicians as it is not a convenient procedure for frequently monitoring disease activity. Fecal calprotectin levels are surrogate markers of colonoscopy as they exhibit a significant correlation with endoscopic activity score, such as the Mayo endoscopic subscore (MES) and Ulcerative Colitis Endoscopic Index of Severity [7–10]. Fecal calprotectin values represent their concentration in stool samples, and they may be affected by inflammatory burden and disease extent. To date, few studies have evaluated the usefulness of fecal biomarkers with respect to the disease extent of UC. In this study, we evaluated fecal biomarkers as surrogate markers of the MES for the extent of disease (total colitis, left-sided colitis, and proctitis).

## Methods

### Patients

A total of 133 UC patients, who received a colonoscopy (total 162 cases) within 30 days of a fecal biomarker test at Kyorin University Hospital from February 2017 to January 2019, were enrolled in this observational study. Patients for whom medications were changed between the time of colonoscopy and fecal biomarker test were excluded. The diagnosis of UC was on the basis of established clinical, radiographic, endoscopic, and histopathologic criteria. Patient characteristics were obtained from medical records. UC clinical disease activity was assessed by the Lichtiger Index (LI), which is composed of the following metrics: the number of daily bowel movements, entity of abdominal pain and tenderness, use of antidiarrheal drugs, blood in stool samples, general well-being, fecal incontinence, and nocturnal diarrhea [11]. In this scoring system, a higher score indicates more severe disease (score range 0–21). Clinical remission was defined as an LI < 4 for patients with UC.

### Measurement of fecal calprotectin, fecal immunochemical test, and fecal lactoferrin

Stool samples were collected within 2 days of measurement and stored at –20 °C until measurement. Fecal calprotectin (FC), fecal immunochemical test for hemoglobin (FIT), and fecal lactoferrin (FL) were measured by the colloidal gold agglutination method using an automatic clinical chemistry analyzer (Hemo Techt NS-Prime; Alfresa-Pharma Corporation, Osaka, Japan) [12].

### Endoscopic assessment

UC patients received a polyethylene glycol-based electrolyte solution for bowel preparation prior to colonoscopy. Colonoscopy was performed by four experienced endoscopists. The interval between colonoscopy and the fecal biomarker test was less than 30 days. Endoscopic disease activity was assessed by two trained endoscopists and was blindly scored according to the MES [13] (Additional file 3: Table 1). The MES is scored on the basis of endoscopic findings on the part of the colon with the most severe inflammation. Endoscopic remission was defined as MES ≤ 1.

### Statistical analysis

Spearman’s rank correlation coefficient was used to analyze the correlation between fecal biomarkers (FC, FIT, and FL) and the MES or LI. Receiver operating characteristic (ROC) analysis was performed to assess the usefulness of these biomarkers analyzed in establishing cutoff values. The criterion for statistical significance was set at *p* < 0.05. GraphPad Prism ver. 8.4.2 software (GraphPad Software, San Diego, CA, USA) was used for the statistical analyses.

## Results

### Patients characteristics

Patients (n = 133) who fulfilled the criteria for UC were enrolled, and colonoscopies were performed with fecal biomarker examination a total of 162 times. There were no adverse events related to colonoscopy in any of the 133 patients. According to the Montreal classification [14, 15], 61 patients (46%) were diagnosed with pancolitis, 48 patients (36%) with left-sided colitis, and 24 patients (18%) with proctitis. The total number of colonoscopies included 79 (49%) for pancolitis, 58 (36%) for left-sided colitis, and 25 (15%) for proctitis. The number of patients with clinical activity (LI ≥ 4) was 25 (42%), 19 (39%), and two (13%) for pancolitis, left-sided colitis, and proctitis, respectively. The number of patients with MES ≥ 2 was 28 (36%) for pancolitis, 25 (43%) for left-sided colitis, and five (20%) for proctitis, respectively. With respect to medications for UC, there were no proctitis patients who received systemic corticosteroid, immunomodulators, oral tacrolimus, tofacitinib, or biologics (Table 1).

**Table 1:** Patient characteristics according to extent of disease

Extent of disease	Pancolitis	Left-sided colitis	Proctitis
Patients (total 133)	61 (46%)	48 (36%)	24 (18%)
Age at the endoscopy (year), median (IQR)	37 (27–51)	46 (33–55)	44 (37–57)
Total number of endoscopies (total 162)	79 (49%)	58 (36%)	25 (15%)
Clinical activity (total 125)			
Remission stage (LI < 4) (total 79)	35 (58%)	30 (61%)	14 (88%)
Active stage (LI ≧ 4) (total 46)	25 (42%)	19 (39%)	2 (13%)
Colonoscopy findings (maximum index in the colorectum)			
MES 0 (total 61)	33 (41%)	17 (29%)	11 (44%)
MES 1 (total 43)	18 (23%)	16 (28%)	9 (36%)
MES 2 (total 45)	20 (26%)	21 (36%)	4 (16%)
MES 3 (total 13)	8 (10%)	4 (7%)	1 (4%)
Concomitant medications			
Oral 5-ASA	68 (85%)	53 (91%)	10 (40%)
Topical 5-ASA	5 (6%)	7 (12%)	11 (44%)
Oral steroids	4 (5%)	1 (2%)	0
Topical steroids	1 (1%)	6 (10%)	5 (20%)
Immunomodulators	24 (3%)	14 (24%)	0
Tacrolimus	0	1 (2%)	0
Infliximab	16 (2%)	6 (10%)	0
Adalimumab	7 (9%)	2 (3%)	0
Golimumab	2 (3%)	1 (2%)	0
Tofacitinib	1 (1%)	0	0

### Correlation between the MES and fecal biomarkers

**Fig. 1:**
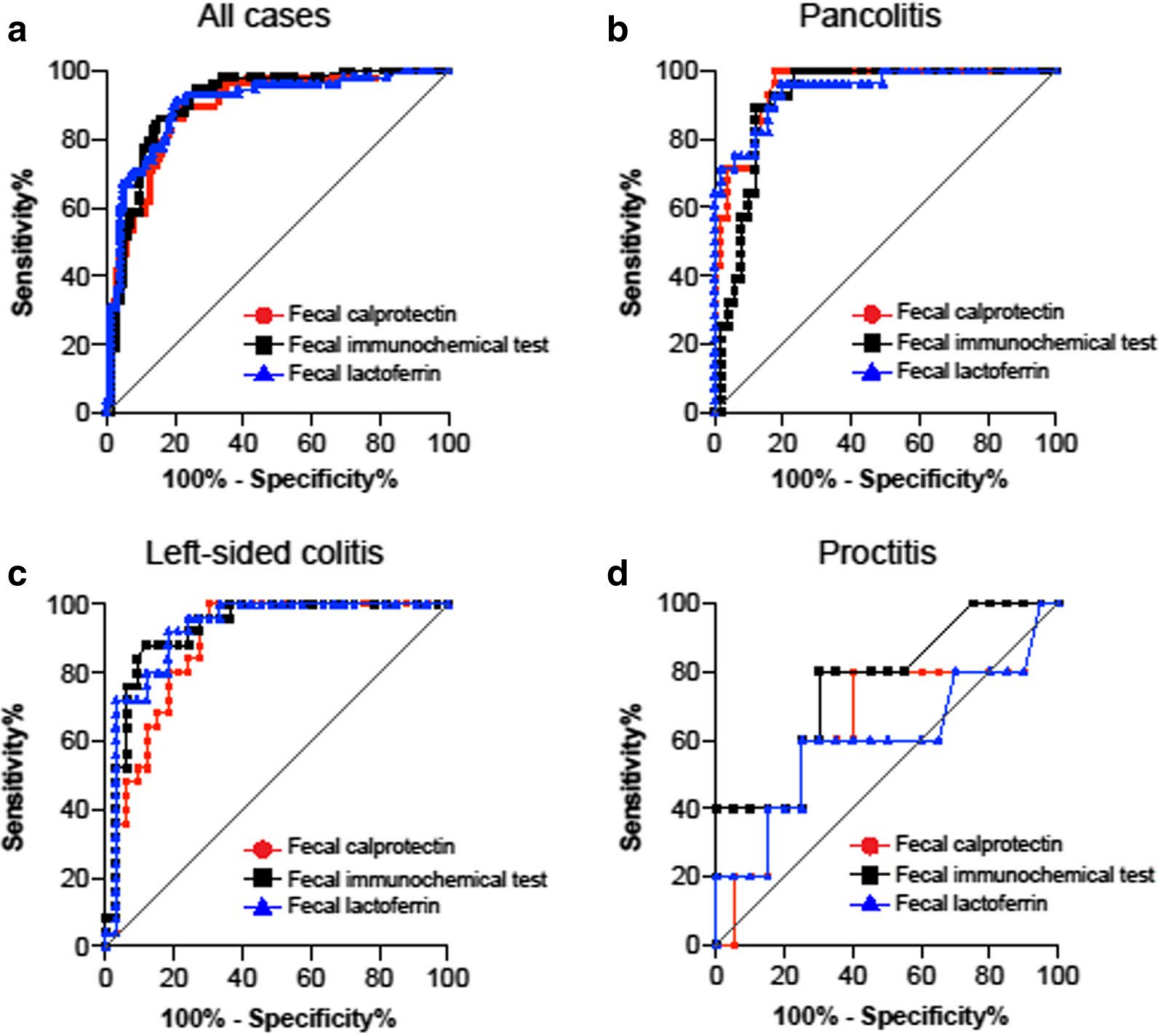
ROC curves for fecal biomarkers (FC, FIT, or FL); MES 0, 1 vs. MES 2, 3. **a** All cases, **b** pancolitis, **c** Left-sided colitis, **d** proctitis. The AUC was lower in the proctitis group for all fecal biomarkers compared with the other subgroups. *ROC* receiver operating characteristic, *FC* fecal calprotectin, *FIT* fecal immunochemical test for hemoglobin, *FL* fecal lactoferrin, *MES* mayo endoscopic score, *AUC* area under the curve

The correlation between the MES and fecal biomarkers was determined. Overall, all three fecal biomarkers (FC, FIT, and FL) were significantly correlated with the MES. The Spearman’s rank correlation coefficients were 0.656, 0.7113, and 0.6943 for FC, FIT, and FL, respectively (Additional file 1: Fig. 1a and Table 2).

**Table 2:** Spearman’s rank correlations between fecal biomarkers and Mayo endoscopic subscore by inflammation location

	FC	FIT	FL
All cases (n = 162)	0.656 *p* < 0.001	0.711 *p* < 0.001	0.694 *p* < 0.001
Pancolitis (n = 79)	0.752 *p* < 0.001	0.693 *p* < 0.001	0.762 *p* < 0.001
Left-sided colitis (n = 52)	0.663 *p* < 0.001	0.784 *p* < 0.001	0.737 *p* < 0.001
Proctitis (n = 25)	0.247 *p* = 0.235	0.545 *p* = 0.005	0.296 *p* = 0.150

*FC* fecal calprotectin, *FIT* fecal immunochemical test for hemoglobin, *FL* fecal lactoferrin

Furthermore, to evaluate the association of disease extent with fecal biomarker values, we determined the correlations relative to the individual disease extent subgroups (pancolitis, left-sided colitis, proctitis). For pancolitis and left-sided colitis, all fecal biomarkers were significantly correlated with the MES. The Spearman’s rank correlation coefficient was 0.752 (FC), 0.693 (FIT), and 0.761 (FL) for pancolitis, whereas coefficients of 0.663 (FC), 0.784 (FIT), and 0.737 (FL) were observed for left-sided colitis (Additional file 1: Fig. 1b, c, and Table 2). In contrast, all fecal biomarkers correlated to a lesser extent with the MES for proctitis (Spearman’s rank correlation coefficient was 0.247, 0.545, and 0.296 for FC, FIT, and FL, respectively) (Additional file 1: Fig. 1d and Table 2).

### Correlation between the LI and fecal biomarkers

**Fig. 2:**
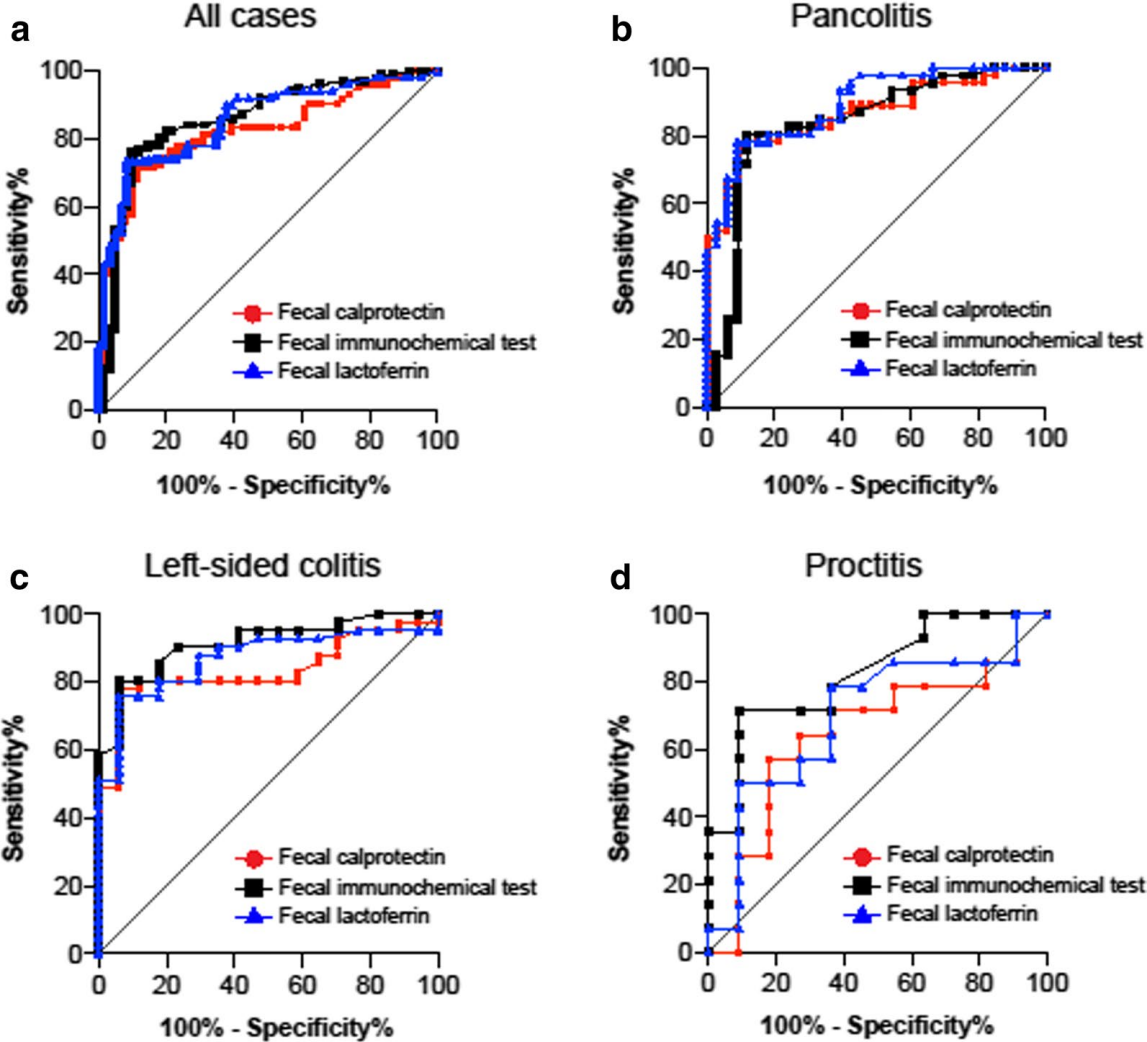
ROC curves for fecal biomarkers (FC, FIT, or FL); MES 0 vs. MES 1, 2, 3. **a** All cases, **b** pancolitis, **c** left-sided colitis, **d** proctitis. All groups, except FC and FL in the proctitis group, were able to distinguish MES 0 with a high AUC. *ROC* Receiver operating characteristic, *FC* fecal calprotectin, *FIT* fecal immunochemical test for hemoglobin, *FL* fecal lactoferrin, *MES* mayo endoscopic score, *AUC* area under the curve

In 125 of 162 cases in which the LI was obtained simultaneously, we evaluated the correlation between fecal biomarker values and the LI. In all cases, the fecal biomarkers were statistically significantly correlated with the LI (Spearman’s rank correlation coefficients were 0.532, 0.531, and 0.528 for FC, FIT, and FL, respectively), but these values were less compared with those of the MES (Additional file 2: Fig. 2a and Table 3). Significant correlations were also observed in the pancolitis and left-sided colitis subgroups. The Spearman’s rank correlation coefficients were 0.465 (FC), 0.397 (FIT), and 0.444 (FL) for pancolitis, and 0.674 (FC), 0.698 (FIT), and 0.679 (FL) for left-sided colitis (Additional file 2: Fig. 2b, c and Table 3). However, the correlations between the LI and fecal biomarkers were not statistically significant for proctitis (Additional file 2: Fig. 2d and Table 3).

**Table 3:** Spearman’s rank correlations between fecal biomarkers and Lichtiger Index by inflammation location

	FC	FIT	FL
All cases (n = 125)	0.532 *p* < 0.001	0.531 *p* < 0.001	0.528 *p* < 0.001
Pancolitis (n = 50)	0.465 *p* = 0.002	0.397 *p* = 0.002	0.444 *p* < 0.001
Left-sided colitis (n = 49)	0.674 *p* < 0.001	0.698 *p* < 0.001	0.679 *p* < 0.001
Proctitis (n = 16)	0.191 *p* = 0.473	0.368 *p* = 0.160	0.133 *p* = 0.620

*FC* fecal calprotectin, *FIT* fecal immunochemical test for hemoglobin, *FL* fecal lactoferrin

### Estimating endoscopic remission with FC, FIT, and FL biomarkers

To estimate endoscopic remission (MES ≤ 1), ROC analysis was performed for each fecal biomarker, and a cutoff value was established for each marker to distinguish MES 0, 1 from MES 2, 3, as determined by the Youden Index. In all cases, all fecal biomarkers could distinguish MES 0, 1 from MES 2, 3 using a high area-under-the-curve (AUC) value (FC: AUC = 0.891, cutoff 298 mg/kg; FIT: AUC = 0.908, cutoff 198 ng/ml; FL: AUC = 0.903, cutoff 96 mg/kg) (Fig. 1a and Table 4). However, in a subgroup analysis of disease extent, AUC was lower in the proctitis group for all fecal biomarkers compared with the other subgroups (Fig. 1b-d and Table 4). For FC, the sensitivity was high in all subgroups, including proctitis (all cases: 0.897, pancolitis: 1.000, left-sided colitis: 1.000, proctitis: 0.800). FC was also indicative of a high negative predictive value (NPV) (all cases: 0.931, pancolitis: 1.000, left-sided colitis: 1.000, proctitis: 0.923). FIT indicated high sensitivity (all cases: 0.862, pancolitis: 0.893, left-sided colitis: 0.880, proctitis: 0.800) and high NPV (all cases: 0.917, pancolitis: 0.938, left-sided colitis: 0.961, proctitis: 0.933). Regarding FL, all subgroups, except for the proctitis group, exhibited high sensitivity (all cases: 0.914, pancolitis: 0.964, left-sided colitis: 0.920, proctitis: 0.600) and high NPV (all cases: 0.943, pancolitis: 0.976, left-sided colitis: 0.931, proctitis: 0.882) (Table 4).

**Table 4:** Summary of predictive values of fecal biomarkers for endoscopic remission (MES 0, 1 vs. 2, 3)

FC	AUC	Cut-off value	Sensitivity	Specificity	PPV	NPV	Accuracy
All cases	0.891	298	0.897	0.779	0.693	0.931	0.821
Pancolitis	0.951	403	1	0.824	0.757	1	0.886
Left-sided colitis	0.882	142	1	0.697	0.714	1	0.828
Proctitis	0.645	185	0.8	0.6	0.333	0.923	0.64

FIT	AUC	Cut-off value	Sensitivity	Specificity	PPV	NPV	Accuracy
All cases	0.908	195	0.862	0.846	0.758	0.917	0.852
Pancolitis	0.915	283	0.893	0.882	0.807	0.938	0.886
Left-sided colitis	0.926	195	0.88	0.879	0.846	0.906	0.879
Proctitis	0.76	30	0.8	0.7	0.4	0.933	0.72

FL	AUC	Cut-off value	Sensitivity	Specificity	PPV	NPV	Accuracy
All cases	0.903	96	0.914	0.798	0.716	0.943	0.840
Pancolitis	0.946	95	0.964	0.804	0.730	0.976	0.861
Left-sided colitis	0.925	111	0.92	0.818	0.793	0.931	0.862
Proctitis	0.6	98	0.6	0.75	0.375	0.882	0.72

*FC* fecal calprotectin, *FIT* fecal immunochemical test for hemoglobin, *FL* fecal lactoferrin, *AUC* area under the curve, *PPV* positive predictive value, *NPV* negative predictive value

Next, the ability to discriminate MES 0 from MES ≥ 1 was evaluated. It was possible to discriminate MES 0 with a high AUC (> 0.800) in all groups except for FC and FL in the proctitis group (Fig. 2). The sensitivity and NPV were lower compared with those used for discriminating MES 0, 1 from MES 2, 3 (Table 5).

**Table 5:** Summary of predictive values of fecal biomarkers for endoscopic remission (MES 0 vs. 1, 2, 3)

FC	AUC	Cut-off value	Sensitivity	Specificity	PPV	NPV	Accuracy
All cases	0.821	290	0.713	0.885	0.911	0.651	0.778
Pancolitis	0.866	344	0.761	0.909	0.921	0.732	0.823
Left-side colitis	0.836	192	0.781	0.941	0.970	0.64	0.828
Proctitis	0.649	270	0.571	0.818	0.8	0.6	0.68

FIT	AUC	Cut-off value	Sensitivity	Specificity	PPV	NPV	Accuracy
All cases	0.858	45	0.762	0.902	0.928	0.696	0.815
Pancolitis	0.835	42	0.804	0.879	0.902	0.763	0.835
Left-side colitis	0.913	46	0.805	0.941	0.971	0.667	0.845
Proctitis	0.825	25	0.714	0.909	0.909	0.714	0.8

FL	AUC	Cut-off value	Sensitivity	Specificity	PPV	NPV	Accuracy
All cases	0.855	79	0.733	0.918	0.937	0.675	0.802
Pancolitis	0.894	79	0.783	0.909	0.923	0.75	0.835
Left-side colitis	0.870	65	0.756	0.941	0.969	0.615	0.810
Proctitis	0.698	98	0.5	0.909	0.875	0.588	0.68

*FC* fecal calprotectin, *FIT* fecal immunochemical test for hemoglobin, *FL* fecal lactoferrin, *AUC* area under the curve, *PPV* positive predictive value, *NPV* negative predictive value

## Discussion

In this study, the most important finding was that the extent of disease can influence the correlation between fecal biomarker values and the MES. The value of each fecal biomarker was presented as its concentration in the stool (i.e., µg/g for calprotectin). Therefore, the results suggest that the value of fecal biomarkers has different meanings between patients with weak extensive inflammation and patients exhibiting strong localized inflammation. We analyzed the correlation of three fecal biomarkers with clinical activity (LI) and endoscopic activity in the most severe part (MES) for each disease extent subgroup on the basis of the Montreal classification. Consistent with previous reports [7, 16, 17], our results demonstrated that fecal biomarkers exhibit better correlation with the MES compared with the LI in most patients and disease extent subgroups.

With respect to the expected role of stool biomarkers as surrogate markers of endoscopy, our study revealed several interesting findings. When we analyzed the correlation of these biomarkers with the MES for each disease subgroup, the results indicated that the proctitis group exhibited a poor correlation compared with the other groups. Until now, there are few reports of fecal biomarkers that have been associated with the extent of disease. In a small study, Roseth et al. [18] showed that in histologically active disease, total colitis FC levels were lower compared with that of proctitis and left-sided colitis. Recently, two reports indicated that the degree of disease affects fecal biomarkers. Sonoyama et al. [19] observed a correlation between endoscopic findings and FC, in addition to blood biomarkers. In this report, the correlation of biomarker levels with the MES was calculated and compared for each disease extent subgroup. FC has been shown to exhibit a significant correlation with endoscopic activity in all disease subgroups, but the correlation was lower for the proctitis type (r = 0.54) compared with the left-colitis (r = 0.75) and total-colitis (r = 0.78) types. Naganuma et al. [20] reported an association between two fecal biomarkers, FC and FIT, and endoscopic activity. Interestingly, the median FC level was lower in patients with proctitis compared with patients experiencing left-sided or total colitis, but the median FIT level was independent of disease extent. These results imply that fecal biomarkers may be interpreted differently depending on the extent of disease, suggesting the possibility that cutoff values are different for each disease subgroup and each fecal biomarker. In our study, as FC, FIT, and FL were measured using the same samples at the same time, we do not have to consider bias resulting from sample differences.

It is important to be able to distinguish endoscopic remission as a surrogate marker for colonoscopy. In all cases, fecal biomarkers were able to distinguish not only MES 0, 1 and MES 2, 3 but also MES 0 and MES 1, 2, 3, although these assessments were varied among each disease extent group. Furthermore, the AUC tended to be lower in the proctitis group. These findings suggest that fecal biomarkers should be used in clinical practice by considering the following points: (1) in proctitis, a correlation with MES is lower, (2) estimating endoscopic remission is more challenging in proctitis compared with other disease types, and (3) the cutoff value may differ depending on disease extent.

The reason for the decreased correlation with fecal biomarkers and the MES in the proctitis group may be related to the shorter distance from the anus and stool retention time. Also, patients with proctitis often do not experience diarrhea, and blood and mucus adhere to the surface of stool resulting in variability in the measured values.

The role of fecal biomarkers should be considered on the basis of two factors: identifying those who have endoscopic activity and selecting patients who have endoscopic remission to avoid unnecessary examination and treatment enhancement. The former is indicated by sensitivity and the latter is indicated by NPV. All these fecal biomarkers were considered to have sufficient potential in these respects. We also attempted to distinguish between MES 0 and MES ≥ 1. For FC and FL, it was possible to distinguish subgroups with high AUC, except for proctitis. Interestingly, FIT was the best at discriminating MES 0 in proctitis; however, NPV was not reliable overall. These results suggest that the cutoff value for fecal biomarkers may change depending on disease extent and the purpose of their use.

There are several limitations to our study. The data were obtained from a single facility. The fecal biomarker measurement method in our study was based on the colloidal gold agglutination method and cannot be directly compared with the results of other reports using different methods. It needs to be noted that the cutoff value of FC differs between measurement kits, and the criteria of FC for endoscopic remission have not been established. Using this method, however, we obtained data for three different fecal biomarkers simultaneously from the same sample. Therefore, the comparison among the three fecal biomarkers was likely more reliable. As the LI could not be obtained in 37 subjects, this could be a bias in this study. Nonetheless, we believe that the characteristics of the LI can be an advantage in this study; the LI was originally developed for assessing the therapeutic response, even in the short term, and has a wide range of scores that are useful in detecting changes in clinical activity [11]. Among the 37 subjects, the MES was 0, 1, 2, and 3 for 15, 15, five, and two subjects, respectively. The number of proctitis patients was relatively small, and the number of patients with MES 3 was low among the cohort of patients, especially in proctitis patients. Patients with proctitis may be less likely to undergo endoscopy compared with patients experiencing pancolitis or left-sided colitis. This is quite possible when considering the burden of endoscopy or the risk of colitic cancer. In addition, it is considered that fecal biomarkers have little clinical significance in patients who have already experienced gross bloody stools, and therefore, the number of patients with MES 3 was relatively low. In fact, there were very few proctitis patients with MES 3 during our daily clinical practice. The difference in the number of subjects and the observed disease activity (particularly endoscopic severity) between proctitis vs. pancolitis/left-sided colitis was an unavoidable limitation in this real-world data-based analysis. Although we employed the MES because it is widely used for assessing UC activity and the definition of endoscopic remission (MES 0, 1) is accepted, given that the MES is based on disease areas with the most severe inflammation, it is a future challenge to determine what kind of scoring system will be the most suitable for a study considering the extent of the inflammation.

In conclusion, for the practical use of fecal biomarkers in UC patients, it is necessary to consider the extent of disease. In particular, patients with proctitis exhibit a low correlation between stool biomarkers and endoscopic findings, resulting in a reduced ability to estimate endoscopic remission, with the exception of FIT.

## Supplementary Information

**Additional file 1: Figure 1**. Spearman’s rank correlation between MES vs. FC, FIT, and FL. (a) All cases, (b) Pancolitis, (c) Left-sided colitis, (d) Proctitis. For proctitis, the correlation coefficient was 0.247 (FC), 0.545 (FIT), and 0.296 (FL). All fecal biomarkers exhibited a lower correlation with the MES. MES, Mayo endoscopic score; FC, Fecal calprotectin; FIT, Fecal immunochemical test for hemoglobin; FL, Fecal lactoferrin.

**Additional file 2: Figure 2**. Spearman’s rank correlation between LI vs. FC, FIT, and FL. (a) All cases, (b) Pancolitis, (c) Left-sided colitis, (d) Proctitis. For proctitis, the correlation coefficient was 0.191 (FC), 0.368 (FIT), and 0.133 (FL). The correlation was not statistically significant, nor were the correlations with LI. LI, Lichtiger Index. FC, Fecal calprotectin; FIT, Fecal immunochemical test for hemoglobin; FL, Fecal lactoferrin.

**Additional file 3: Table 1**. Mayo Endoscopic Subscore (MES)

## Data Availability

The datasets used and analyzed during this study are available from the corresponding author upon reasonable request.

## Abbreviations

ASA: Aminosalicylic acid
AUC: Area under the curve
FC: Fecal calprotectin
FIT: Fecal immunochemical test for hemoglobin
FL: Fecal lactoferrin
IQR: Interquartile range
LI: Lichtiger Index
NPV: Negative predictive value
MES: Mayo endoscopic subscore
PPV: Positive predictive value
ROC: Receiver operating characteristic
UC: Ulcerative colitis
