# Supplementary material for: Extent of disease affects the usefulness of fecal biomarkers in ulcerative colitis
Source: BMC Gastroenterol. 2021 May 1;21:197. doi: 10.1186/s12876-021-01788-4 (PMC8088576; doi:10.1186/s12876-021-01788-4)

Extent of disease affects the usefulness of fecal biomarkers in ulcerative colitis

Authors:

Akihito Sakuraba[1], Nobuki Nemoto[1], Noritaka Hibi[1], Ryo Ozaki[1], Sotaro Tokunaga[1], Oki Kikuchi[1], Shintaro Minowa[1], Tatsuya Mitsui[1], Miki Miura[1], Daisuke Saito[1], Mari Hayashida[1], Jun Miyoshi[1], Minoru Matsuura[1], Masayoshi Yoneyama[2], Hiroaki Ohnishi[3], Tadakazu Hisamatsu[1]＊

Affiliation:

1) Department of Gastroenterology and Hepatology, Kyorin University School of Medicine, Tokyo, Japan
2) Department of Clinical Laboratory, Kyorin University Hospital, Tokyo, Japan.
3) Department of Laboratory Medicine, Kyorin University School of Medicine, Tokyo, Japan.

＊Corresponding author:

Tadakazu Hisamatsu, M.D., Ph.D., FACG, AGAF

Department of Gastroenterology and Hepatology,

Kyorin University School of Medicine.

6-20-2 Shinkawa, Mitaka-shi, Tokyo 181-8611, Japan

Tel.: +81-422-47-5511 (ext. 5279)

Fax: +81-422-44-0655

E-mail: thisamatsu@ks.kyorin-u.ac.jp

Running title: Disease extent and fecal biomarkers in UC

Supplemental Figure 2

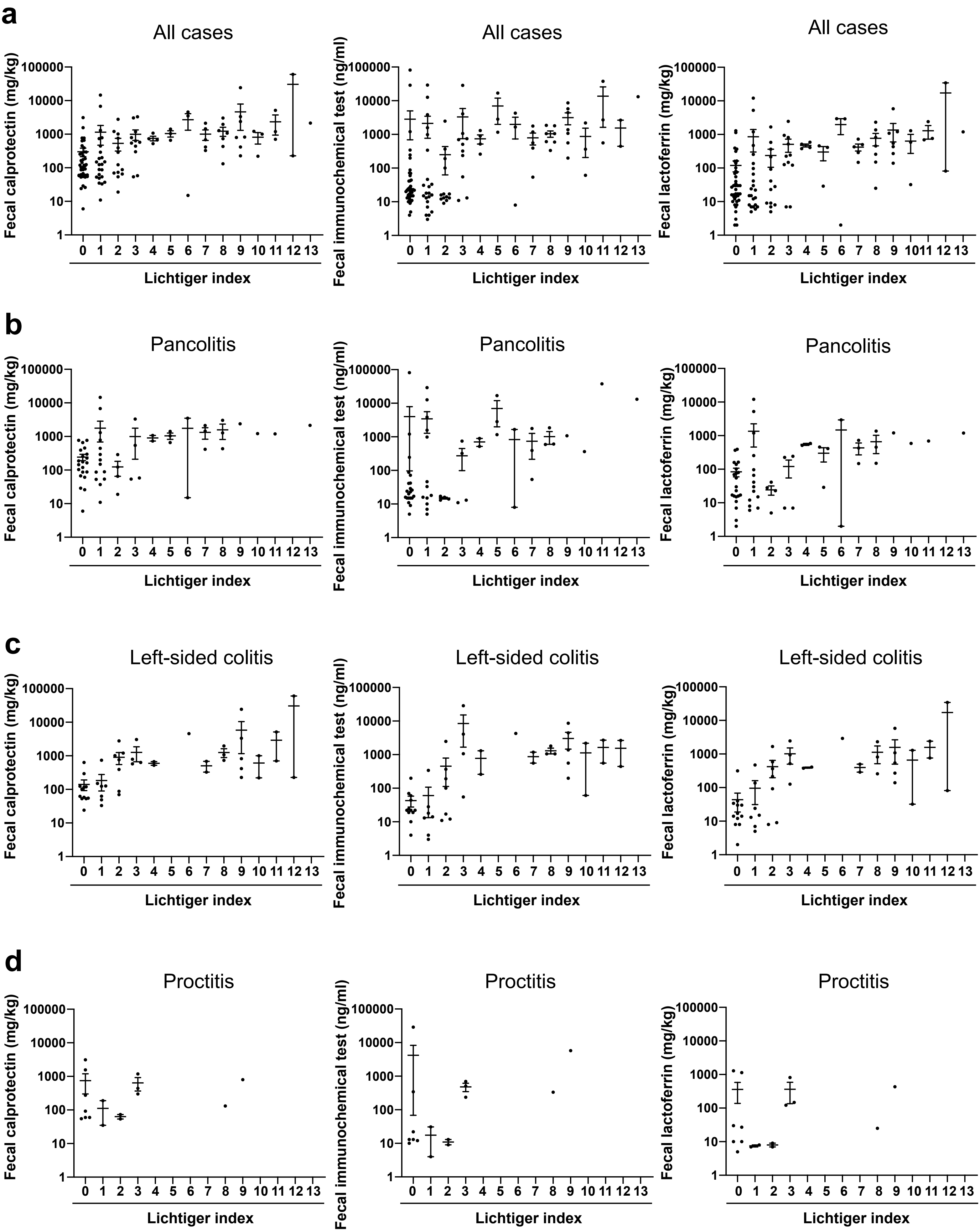

Supplement: Supplementary file 2 — Additional file 2: Figure 2. Spearman’s rank correlation between LI vs. FC, FIT, and FL. (a) All cases, (b) Pancolitis, (c) Left-sided colitis, (d) Proctitis. For proctitis, the correlation coefficient was 0.191 (FC), 0.368 (FIT), and 0.133 (FL). The correlation was not statistically significant, nor were the correlations with LI. LI, Lichtiger Index. FC, Fecal calprotectin; FIT, Fecal immunochemical test for hemoglobin; FL, Fecal lactoferrin. [file 12876_2021_1788_MOESM2_ESM.pdf]
