# Supplementary material for: Extent of disease affects the usefulness of fecal biomarkers in ulcerative colitis
Source: BMC Gastroenterol. 2021 May 1;21:197. doi: 10.1186/s12876-021-01788-4 (PMC8088576; doi:10.1186/s12876-021-01788-4)
Supplement: Supplementary file 3 — Additional file 3: Table 1. Mayo Endoscopic Subscore (MES) [file 12876_2021_1788_MOESM3_ESM.pdf]

Extent of disease affects the usefulness of fecal biomarkers in ulcerative colitis

Authors:

Akihito Sakuraba[1], Nobuki Nemoto[1], Noritaka Hibi[1], Ryo Ozaki[1], Sotaro Tokunaga[1], Oki Kikuchi[1], Shintaro Minowa[1], Tatsuya Mitsui[1], Miki Miura[1], Daisuke Saito[1], Mari Hayashida[1], Jun Miyoshi[1], Minoru Matsuura[1], Masayoshi Yoneyama[2], Hiroaki Ohnishi[3], Tadakazu Hisamatsu[1]＊

Affiliation:

1) Department of Gastroenterology and Hepatology, Kyorin University School of Medicine, Tokyo, Japan
2) Department of Clinical Laboratory, Kyorin University Hospital, Tokyo, Japan.
3) Department of Laboratory Medicine, Kyorin University School of Medicine, Tokyo, Japan.

＊Corresponding author:

Tadakazu Hisamatsu, M.D., Ph.D., FACG, AGAF

Department of Gastroenterology and Hepatology,

Kyorin University School of Medicine.

6-20-2 Shinkawa, Mitaka-shi, Tokyo 181-8611, Japan

Tel.: +81-422-47-5511 (ext. 5279)

Fax: +81-422-44-0655

E-mail: thisamatsu@ks.kyorin-u.ac.jp

Running title: Disease extent and fecal biomarkers in UC

Supplemental Table 1. Mayo Endoscopic Subscore (MES)

Score	Disease Activity	Endoscopic Features (Descriptors)
0	Normal or inactive	None
1	Mild	Erythema, decreased vascular pattern, mild friability
2	Moderate	Marked erythema, absent vascular pattern, friability, erosions
3	Sever	Spontaneous bleeding, ulceration
